# Association of folate metabolism-related enzymes (MTHFR, MYD88, and TP53) and their single nucleotide polymorphisms with breast cancer susceptibility in women from Southwest China: a Bayesian network approach

**DOI:** 10.3389/fonc.2025.1560776

**Published:** 2025-09-25

**Authors:** Xingxu Li, Yunyu Gong, Yuxin Zhao, Xianshu Kong, Yingbo Liu, Qunshan Liu, Yunjiao Zhang, Zhen Li

**Affiliations:** ^1^ Yunnan College of Finance and Economics, Kunming, Yunnan, China; ^2^ Yunnan University of Finance and Economics, Kunming, Yunnan, China; ^3^ Yunnan Key Laboratory of Breast Cancer Precision Medicine, Second Department of Breast Surgery, Yunnan Cancer Hospital, The Third Affiliated Hospital of Kunming Medical University, Peking University Cancer Hospital Yunnan, Kunming, Yunnan, China; ^4^ Department of Breast Surgery, Third People’s Hospital of Honghe Prefecture, Cancer Hospital of Honghe Prefecture, Honghe, China; ^5^ Kunming Medical University Haiyuan College, Kunming, China

**Keywords:** breast cancer, SNPs (single nucleotide polymorphism), Bayesian networks (BNs), folate(folic acid), MyD88, TP53

## Abstract

**Background:**

Breast cancer remains one of the most prevalent malignant tumors affecting women globally. Genetic factors are significant contributors to its pathogenesis. Single nucleotide polymorphisms (SNPs), as a common form of genetic variation, have garnered considerable attention in recent years. However, most studies have predominantly focused on associations between individual loci and breast cancer susceptibility, while the complex interactions among multiple loci across different genes remain insufficiently explored.

**Methods:**

To analyze high-dimensional multi-locus variables, chi-square test and random forests were employed. Bayesian networks, a sophisticated statistical model, were used to investigate SNP interactions across multiple genes and to construct a comprehensive genetic susceptibility model for female breast cancer.

**Results:**

The study analyzed 980 samples, comprising 490 breast cancer patients and 490 controls. Key intergenic genotypes were identified involving SNPs in TP53 (rs1042522), MTHFR (rs1801133), MTHFR (rs56221660), MTRR (rs1801394), MTR-A2756G (rs1805087), MYD88 (rs7744), and rs7851696. These interactions were associated with a significant increase in breast cancer prevalence, rising from 48.2% in the original data to 99% under the largest posterior probability combination. External validation further demonstrated a breast cancer prevalence of 70%, underscoring the robustness of the model.

**Conclusions:**

Interactions among the TP53, MYD88, and folate metabolism-related genes (MTHFR, MTR, and MTRR) may play a critical role in breast cancer susceptibility.

## 1 Introduction

Breast cancer poses a major global health challenge, with 2022 data from the International Agency for Research on Cancer (IARC) reporting 2.3 million new cases, representing 11.6% of all cancer diagnoses, and 666 000 deaths, accounting for 6.9% of all cancer-related fatalities. In 2023, the global incidence reached approximately 47.8 per 100 000 women (1). Advances in medical care and increased emphasis on screening have inadvertently contributed to the observed rise in breast cancer incidence. Several well-established risk factors contribute to breast cancer susceptibility, including early menarche, late menopause, advanced age, frequent childbirth, oral contraceptive use, obesity, and alcohol consumption (2). Genetic factors are also increasingly recognized as critical contributors to breast cancer risk. Mutations in BRCA1 and BRCA2, as well as other genes such as TP53 and PALB2, have been strongly linked to an elevated risk of developing the disease (3).

Treatment strategies for breast cancer encompass surgery, radiation therapy, chemotherapy, endocrine therapy, targeted therapy, and immunotherapy. However, despite advancements, conventional surgical treatment and radiotherapeutic approaches are associated with significant recurrence risks and adverse side effects. Precision medicine approaches, including targeted therapies and immunotherapy, have demonstrated superior efficacy, offering more personalized and precise treatment options. Given the diverse and often uncontrollable nature of breast cancer risk factors, the World Health Organization (WHO) emphasizes early diagnosis, routine screening, and comprehensive health management to improve outcomes (1).

With this in mind, we focused on the genetic underpinnings of female breast cancer by identifying genetic variants associated with the disease. These variants may influence cancer development, disease progression, and therapeutic sensitivity, providing critical insights for the prevention, diagnosis, and treatment of breast cancer in clinical settings. Among the various forms of genetic variation, single nucleotide polymorphisms (SNPs) are the most prevalent. These single-nucleotide variations in DNA sequences among individuals play a key role in genetic diversity and disease susceptibility (4).

The study of SNPs in relation to breast cancer has primarily focused on several well-characterized genetic loci. Folic acid (FA) is an essential B vitamin that must be obtained from dietary sources. It plays a critical role in cellular processes, and its deficiency is implicated in various diseases, including hypertension, cardiovascular disease, neural tube defects, neonatal megaloblastic anemia, and malignant tumors (5–11). Enzymes involved in folate metabolism, such as methylenetetrahydrofolate reductase (MTHFR), methionine synthase (MTR), and methionine synthase reductase (MTRR), are of particular interest due to their roles in oncogenesis and polymorphic loci. These enzymes are integral to DNA synthesis, repair, and methylation processes (12–15), disruptions of which can precipitate carcinogenesis.

Polymorphisms in MTHFR are hypothesized to influence breast cancer susceptibility by altering DNA methylation, homocysteine metabolism, and related pathways. The MTHFR gene, located on human chromosome 1 (1p36.3), spans 11 exons and 10 introns, with a cDNA length of 2 220 bp (16). SNPs such as C677T (rs1801133) and MTRR A66G (rs1801394) have been shown to impact enzyme activity and exhibit strong correlations with breast cancer (17–21). For example, based on MassARRAY and regression analyses, Tao et al. demonstrated an association between the MTRR (rs1801394) locus and increased breast cancer risk (22). Additionally, specific genotypes of these loci have differential effects on breast cancer risk. The TT genotype of rs1801133 in MTHFR significantly increases the risk of breast cancer, whereas the CC genotype of rs9651118 is associated with reduced disease risk and improved survival (23). Knockdown of MTR in tumor cells disrupts folate metabolism, leading to impaired purine synthesis, nucleotide depletion, and reduced tumor growth in both cell culture and xenograft models (24). Furthermore, carriers of the MTRR (A2756G) mutation exhibit an elevated risk of breast cancer (24).

Beyond folate metabolism, other genes, such as myeloid differentiation factor 88 (MYD88) and TP53, are implicated in breast cancer pathogenesis. MYD88, a key promoter of inflammation, fosters an inflammatory microenvironment conducive to carcinogenesis (25). The TP53 gene, a well-known risk factor for breast cancer, influences susceptibility through SNPs in intronic and promoter regions, such as rs1625895 and rs17878362, which alter gene cleavage and transcriptional regulation, substantially elevating cancer risk (26, 27).

Traditional statistical approaches have been the primary methods used to explore the relationship between SNPs and breast cancer. These methods typically assess the significance of individual loci based on *P*-values or evaluate interaction effects between loci to elucidate their combined influence on disease susceptibility. However, such approaches may only partially capture the complex interplay of genetic factors contributing to breast cancer risk in women, with few studies exploring the concerted effects of multiple genes in breast cancer. To address this limitation, advanced statistical models, such as Bayesian networks, offer a robust framework for uncovering complex relationships among polymorphic loci across multiple genes. Bayesian networks, a cornerstone of modern interpretable artificial intelligence, represent conditional relationships through an acyclic graphical structure and a set of probability tables that detail variable dependencies. These models have been widely recognized for their utility in simulating biological systems (28) and have been applied to signal data analysis (29, 30), chromatin construction, and interaction modeling (31). The primary advantage of Bayesian networks over other probabilistic modeling approaches lies in their flexibility: they do not require predefined input and output variables and can be constructed even with limited evidence of associations among the variables of interest (32). Additionally, their graphical representation enables direct interpretation of variable relationships, offering conditional (33).

In summary, this study sequenced 27 single nucleotide loci to investigate polymorphisms associated with breast cancer. Initial analyses, including chi-square testing and random forest (RF) modeling, were conducted to identify relevant loci, guided by existing literature. Modeling analysis loci included rs1042522, rs17884306, rs1801133, rs1801394, rs1805087, rs56221660, rs7744, rs7851696, and rs9651118. Subsequently, the study examined the associations between these loci and breast cancer susceptibility in women, providing new insights into their potential roles in disease development.

## 2 Materials and methods

### 2.1 Materials

This study utilized data from 490 confirmed breast cancer cases, matched with 490 control samples, aged between 20 and 75 years. All participants were long-term residents of Yunnan Province, China, with ancestry spanning at least three generations in the region. Blood samples (1 mL of fasting whole blood) were collected using EDTA anticoagulant tubes. Genomic DNA was extracted using a Promega Whole Blood DNA Extraction Kit. DNA concentration and quality were assessed using a NanoDrop 2000c spectrophotometer, ensuring a minimum concentration of 40 ng/µL. SNPs at loci of interest, including those in MTHFR and other relevant genes, were detected using high-resolution time-of-flight mass spectrometry (TOFMS) biochip systems. This study analyzed the relationship between gene polymorphisms at relevant loci and breast cancer, with additional emphasis on polymorphic loci in immune-related genes. Informed consent was obtained from all participants prior to sample collection and data analysis.

### 2.2 Methods

**Figure 1:**
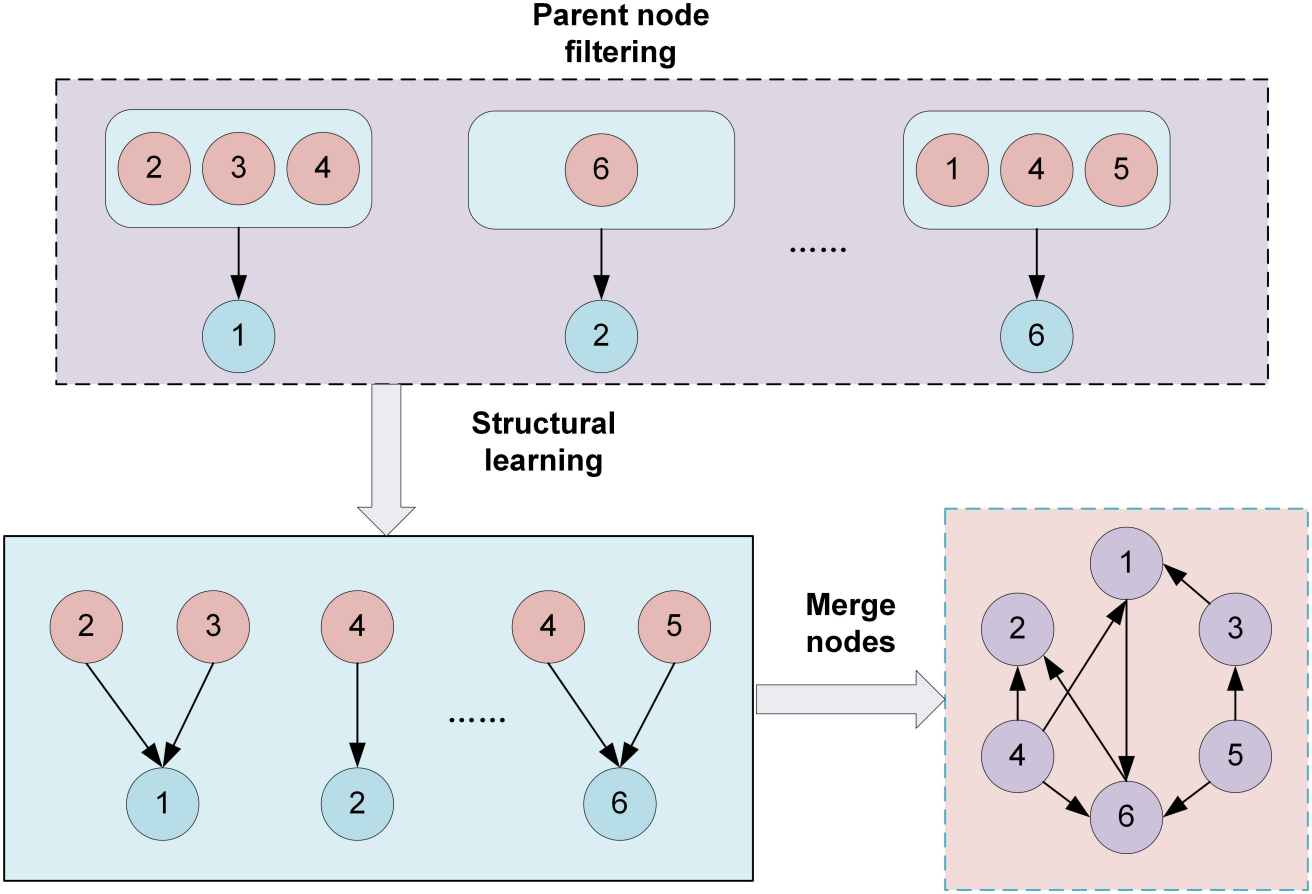
Screen the Bayesian network structure based on the parent node.


Data preprocessing, organization, and analysis were conducted using R Studio. The Hardy-Weinberg equilibrium was determined using the chi-square test. Preliminary exploration of polymorphic sites associated with relevant enzymes was conducted using either a univariate chi-square test or Fisher’s exact test, with a P-value of less than 0.05 considered statistically significant. Polymorphic loci with feature importance scores exceeding 0.05, as determined by the RF algorithm, were extracted. In addition, the study also integrated the breast cancer susceptibility gene loci that have been reported in the literature to evaluate their high-dimensional interactions and their contribution to disease risk. In this paper, a Bayesian network model is adopted to analyze the complex interaction relationship among polymorphisms of different gene loci. The model is constructed through the Bayesian Network Toolbox of the MATLAB platform (FullBNT-1.0.7, RRID: SCR_001622). The adopted Bayesian network modeling method has the following advantages: Firstly, the probabilistic graphical model can visually represent the conditional dependency relationship among various gene loci and quantify its intensity of effect; Secondly, compared with the data encoding process required by traditional statistical methods, this method can retain the original data information more completely. In addition, its modular architecture inherently supports incremental learning and data expansion. In view of the limited sample size of the current research, this paper improves the structure learning algorithm of Bayesian networks. By proposing a network structure construction method based on whether Cramer’s V coefficient belongs to strong correlation and combining expert experience and the K2 algorithm for structure learning, as shown in **Figure 1**. It significantly enhanced the reliability of the model and the credibility of the results under the condition of small samples. Cramer’s V coefficient is denoted as φ. The formula is shown in **Equation 1**.

(1) $$\phi_{c}=\sqrt{\frac{\chi^{2}/n}{\min\left(k-1,r-1\right)}}$$

Here, the Pearson chi-square value,:sample size,:number of rows in the cross table,:number of columns in the cross label.

In a Bayesian network structure, each node corresponds to a random variable. Different nodes represent different states of gene loci in breast cancer patients. Each node is associated with a conditional probability distribution, quantitatively describing the probability of occurrence at that node under the parent node. Directed edges represent causal or dependent relationships, while edges pointing from a parent node to a child node represent direct dependencies. After constructing the network structure through this method, the Bayesian estimation method is adopted for network parameter learning, and the join tree reasoning method is used for posterior inference. When training the data, the training set and the test set are mainly divided in a 7:3 ratio.

## 3 Results

### 3.1 Chi-square test results

The 27 genetic polymorphic loci examined in this study were assessed for Hardy-Weinberg equilibrium, which assumes constant allele and genotype frequencies in the absence of migration, mutation, or selection. All loci were found to conform to the Hardy-Weinberg equilibrium, confirming their suitability for subsequent analyses.

Chi-square tests or Fisher’s exact tests were used to evaluate the distribution of polymorphic loci between breast cancer cases and controls. The *P*-values of these analyses are shown in **Table 1**. Statistically significant differences were observed between the breast cancer and control groups for rs1801133 (*P* = 0.005) and rs56221660 (*P* = 0.049), suggesting a potential association between these SNPs and breast cancer development in women.

**Table 1:** Univariate analysis of breast cancer loci.

Genetic locus	N (%)	Breast cancer		*P*-value	Genetic locus	N (%)	Breast cancer		*P-*value
		Yes	No				Yes	No	
rs1042522				0.880	rs1994798				0.375
C	293 (31.8%)	147 (32.1%)	146 (31.6%)		A	580 (63%)	296 (64.6%)	284 (61.5%)	
CG	439 (47.8%)	215 (46.9%)	224 (48.5%)		G	40 (4.4%)	22 (4.8%)	18 (3.9%)	
G	188 (20.4%)	96 (21.0%)	92 (19.9%)		GA	300 (32.6%)	140 (30.6%)	160 (34.6%)	
rs11559040				0.873	rs2066462				0.550
A	11 (1.1%)	6 (1.3%)	5 (1.1%)		A	6 (0.6%)	4 (0.9%)	2 (0.4%)	
G	725 (78.8%)	363 (79.3%)	362 (78.3%)		G	776 (84.4%)	390 (85.2%)	386 (83.5%)	
GA	184 (21%)	89 (19.4%)	95 (20.6%)		GA	138 (15%)	64 (14.0%)	74 (16.0%)	
rs1537514				0.474	rs2066470				0.369
C	6 (0.6%)	4 (0.9%)	2 (0.4%)		A	6 (0.6%)	4 (0.9%)	2 (0.4%)	
CG	141 (15.5%)	65 (14.2%)	76 (16.4%)		AG	146 (15.9%)	65 (14.2%)	81 (17.5%)	
G	773 (84.1%)	389 (84.9%)	384 (83.1%)		G	768 (83.5%)	389 (84.9%)	379 (82.0%)	
rs1537516				0.474	rs2184227				0.452
A	6 (0.6%)	4 (0.9%)	2 (0.4%)		C	772 (84%)	388 (84.7%)	384 (83.1%)	
G	773 (84.1%)	389 (84.9%)	384 (83.1%)		CT	142 (15.4%)	66 (14.4%)	76 (16.4%)	
GA	141 (15.3%)	65 (14.2%)	76 (16.4%)		T	6 (0.6%)	4 (0.8%)	2 (0.4%)	
rs17884306				0.309	rs2274976				0.492
C	771 (83.8%)	387 (84.5%)	384 (83.1%)		C	774 (84.2%)	389 (84.9%)	385 (83.3%)	
CT	143 (15.5%)	70 (15.3%)	73 (15.8%)		CT	140 (15.2%)	65 (14.2%)	75 (16.2%)	
T	6 (0.6%)	1 (0.2%)	5 (1.1%)		T	6 (0.6%)	4 (0.9%)	2 (0.4%)	
rs1800629				0.900	rs3737964				0.842
A	6 (0.6%)	3 (0.7%)	3 (0.6%)		C	724 (78.7%)	363 (79.3%)	361 (78.1%)	
G	817 (88.8%)	409 (89.3%)	408 (88.3%)		T	11 (1.2%)	6 (1.3%)	5 (1.1%)	
GA	97 (10.6%)	46 (10.0%)	51 (11.0%)		TC	185 (20.1%)	89 (19.4%)	96 (20.8%)	
rs1801131				0.760	rs3737965				0.321
G	29 (3.3%)	14 (3.1%)	15 (3.2%)		A	6 (0.6%)	4 (0.9%)	2 (0.4%)	
GT	283 (30.7%)	136 (29.7%)	147 (31.8%)		G	768 (83.5%)	389 (84.9%)	379 (82.0%)	
T	608 (66.0%)	308 (67.2%)	300 (64.9%)		GA	146 (15.9%)	65 (14.2%)	81 (17.5%)	
rs1801133				0.005	rs3737966				0.763
A	42 (4.6%)	18 (3.9%)	24 (5.2%)		C	40 (4.4%)	21 (4.6%)	19 (4.1%)	
AG	701 (76.2%)	370 (80.8%)	331 (71.7%)		CT	291 (31.6%)	140 (30.6%)	151 (32.7%)	
G	177 (19.2%)	70 (15.3%)	107 (23.2%)		T	589 (64%)	297 (64.8%)	292 (63.2%)	
rs1801394				0.327	rs3737967				0.335
A	536 (58.3%)	278 (60.7%)	258 (55.8%)		A	7 (0.7%)	5 (1.1%)	2 (0.4%)	
AG	327 (35.5%)	153 (33.4%)	174 (37.7%)		AG	140 (15.3%)	64 (14.0%)	76 (16.4%)	
G	57 (6.2%)	27 (5.9%)	30 (6.5%)		G	773 (84%)	389 (84.9%)	384 (83.1%)	
rs1805087				0.079	rs4846048				0.632
A	736 (80%)	366 (79.9%)	370 (80.1%)		A	706 (76.7%)	353 (77.1%)	353 (76.4%)	
G	5 (0.5%)	0 (0.0%)	5 (1.1%)		AG	201 (21.8%)	97 (21.2%)	104 (22.5%)	
GA	179 (19.5%)	92 (20.1%)	87 (18.8%)		G	13 (1.5%)	8 (1.8%)	5 (1.1%)	
rs4968187				0.916	rs56221660				0.049
C	762 (82.8%)	378 (82.5%)	384 (83.1%)		A	753 (81.8%)	387 (84.5%)	366 (79.2%)	
T	4 (0.4%)	2 (0.4%)	2 (0.4%)		AG	161 (17.6%)	67 (14.6%)	94 (20.4%)	
TC	154 (16.8%)	78 (17.0%)	76 (16.4%)		G	6 (0.6%)	4 (0.9%)	2 (0.4%)	
rs6853				0.823	rs72640221				0.636
A	882 (95.9%)	438 (95.6%)	444 (96.1%)		A	7 (0.7%)	4 (0.9%)	3 (0.6%)	
AG	37 (4.1%)	19 (4.2%)	18 (3.9%)		G	774 (84.2%)	390 (85.2%)	384 (83.1%)	
G	1 (1%)	1 (0.2%)	0 (0.0%)		GA	139 (15.1%)	64 (14.0%)	75 (16.2%)	
rs7744				0.269	rs7851696				0.992
A	378 (41%)	200 (43.7%)	178 (38.5%)		G	607 (66.0%)	303 (66.2%)	304 (65.8%)	
G	136 (14.8%)	63 (13.8%)	73 (15.8%)		GT	279 (30.3%)	138 (30.1%)	141 (30.5%)	
GA	406 (44.1%)	195 (42.6%)	211 (45.7%)		T	34 (3.7%)	17 (3.7%)	17 (3.7%)	
rs9651118				0.535					
C	151 (16.4%)	69 (15.1%)	82 (17.8%)						
CT	414 (45%)	208 (45.4%)	206 (44.6%)						
T	355 (38.6%)	181 (39.5%)	174 (37.7%)						

it is indicated that these extreme probabilities reflect characteristics of the sample distribution rather than clinical predictive value.

### 3.2 RF results

To identify additional SNPs with potential relevance, an RF machine learning-based approach was employed. Unlike traditional one-way statistical analyses, the RF model excels in identifying critical features through iterative, data-driven computations. Here, the feature importance of SNPs was calculated by training the RF model which aggregates the importance of all features associated with each SNP. The formula used to compute the cumulative importance of features for each SNP is provided below:


$$I_{i}=\sum_{j}x_{ij}$$

where *i* denotes the index of the SNP, *x* represents the feature importance score, and *j* refers to the features associated with the *i*th SNP. Thus, *j* corresponds to the *j*th feature derived from the *i*th SNP.

**Figure 2:**
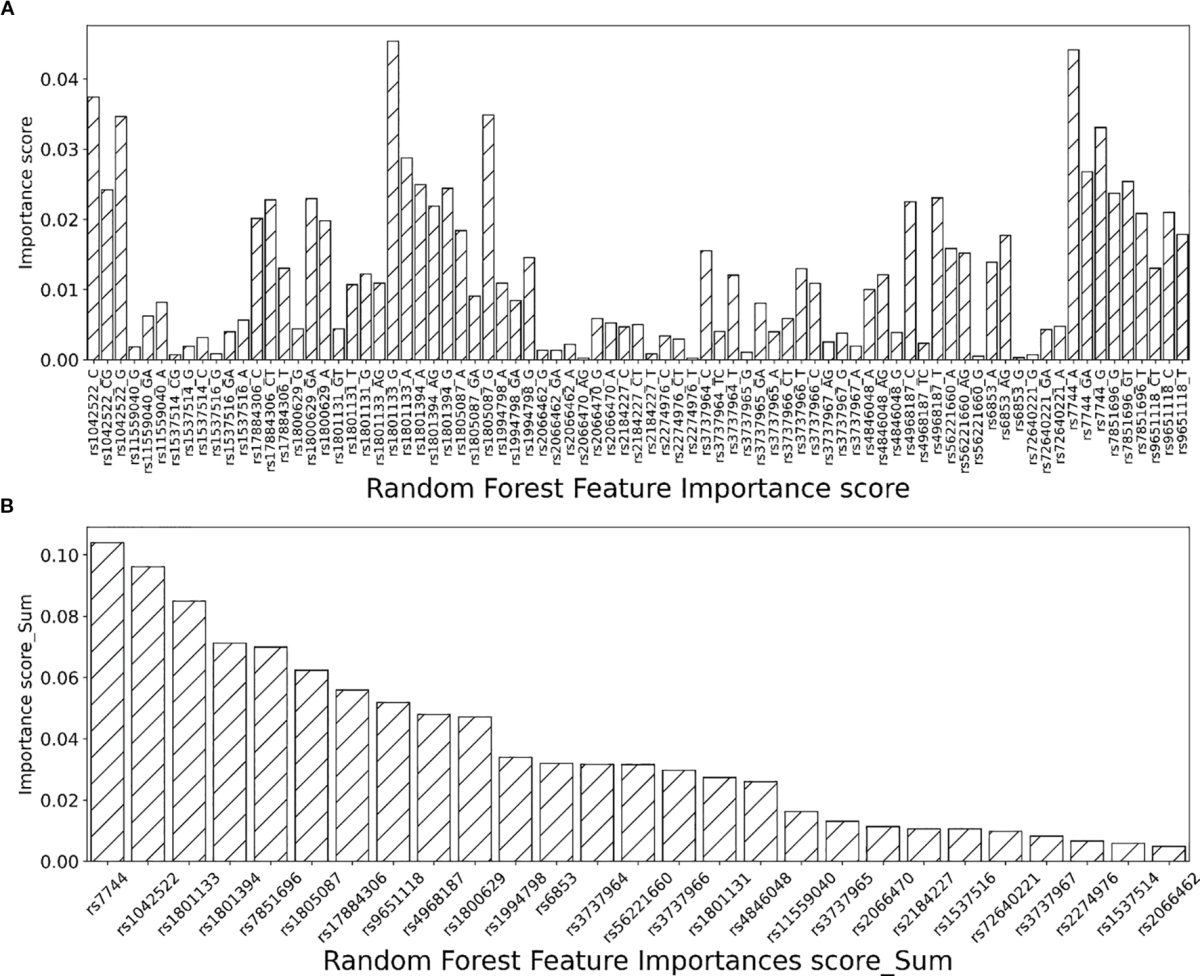
Random forest model variable screening.

Mean decrease impurity (MDI) based on the machine learning model RF is used for importance analysis. Specifically, the importance of features is obtained by calculating the impact of each feature on the observed impurity of each node of the classification tree. Larger values indicate that the feature is more significant. Graph A in **Figure 2** shows the feature importance scores for all 81 features generated from the 27 single nucleotide sites based on the RF model. The importance of the features calculated based on the RF model was calculated by summing the importance of the features generated for each SNP. (**Figure 2B**) illustrates the importance ranking of SNPs based on the RF model. The importance scores ranged from the most important to the least important in the prediction of female breast cancer prevalence. The results showed that rs7744 ranked first in the RF model (**Figure 2B**). And the A genotype carried on the rs7744 locus had a significantly increased risk of breast cancer compared with patients carrying the G or GA genotype (**Figure 2A**). Similarly, the risk level of the remaining gene loci can be clearly seen. Here, the main focus is to extract the features whose sum of feature importance is greater than 0.05 for subsequent analysis. The extracted gene loci are as follows: rs7744, rs1042522, rs1801133, rs1801394, rs7851696, rs1805087, rs17884306, rs9651118.

### 3.3 Bayesian network modeling results

#### 3.3.1 Bayesian network structure learning

**Figure 3:**
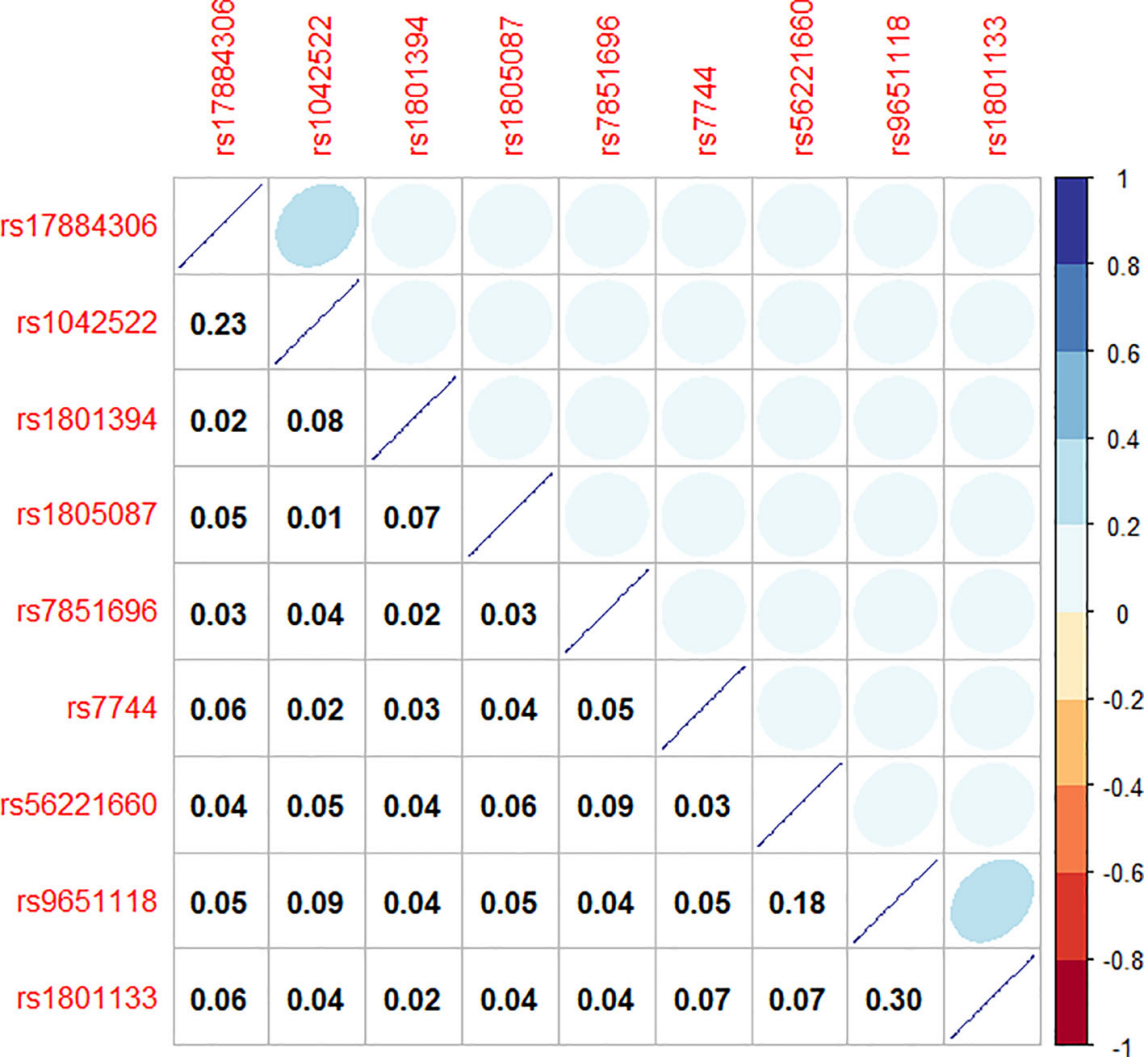
Cramer’s V coefficient heat map.

**Figure 4:**
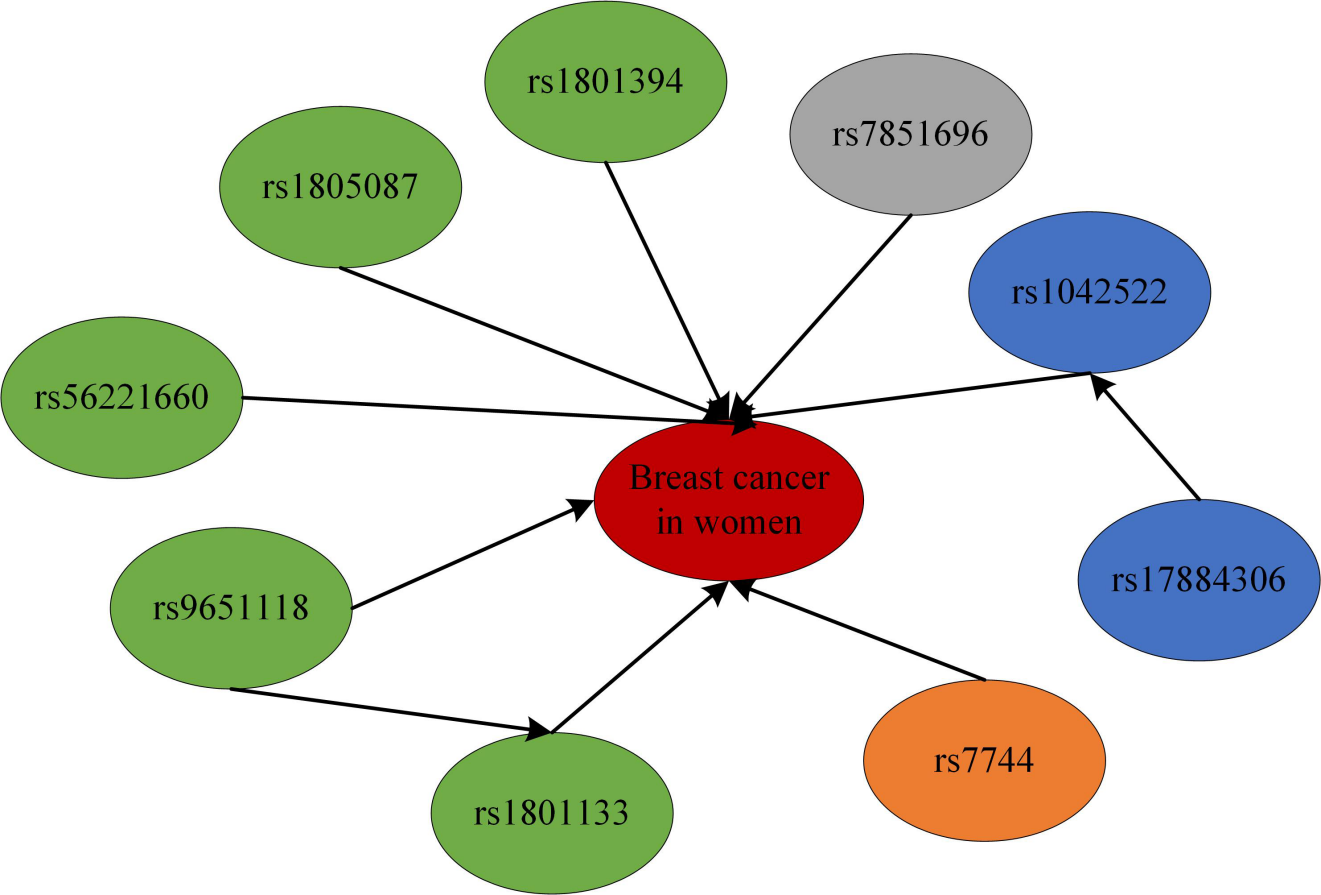
Bayesian network model of breast cancer susceptibility loci.

To model the genetic associations underlying female breast cancer prevalence, Bayesian network structure learning was conducted using SNPs identified through chi-square tests and RF analyses. The Bayesian network structure was constructed using the Strong correlation method based on Cramer’s V coefficient (**Figure 3**), along with expert domain knowledge, resulting in a structure comprising 10 nodes and 10 directed edges, representing the interrelationships among breast cancer SNP loci, with different colors representing different genes (**Figure 4**). The Bayesian network revealed direct correlations between several loci, including rs1042522, rs1801133, rs1801394, rs1805087, rs56221660, rs7744, and rs7851696, and breast cancer susceptibility. Additionally, the network identified indirect associations involving rs17884306 and rs9651118, highlighting their potential involvement in breast cancer pathogenesis. This network provides a comprehensive visualization of the genetic architecture underlying breast cancer susceptibility in women.

#### 3.3.2 Bayesian network parameter learning

To construct an accurate Bayesian network architecture for female breast cancer, Bayesian estimation was employed to learn the parameters associated with each network node. The parameter estimates are summarized in **Table 2**. For specific genotypic combinations of loci, the probabilities of breast cancer were strikingly high. It is important to note that such high-probability data represent the highest-risk combinations observed in the current small sample. For example, when the genotype at loci rs1042522, rs1801133, rs1801394, rs1805087, rs56221660, rs7744, and rs7851696 was [CG A A A A A A G], the probability of developing breast cancer was 99.98%, with a 0.02% chance of remaining disease-free. Similarly, when the genotype was [CG G A A A A G], the probability of developing breast cancer was 99.98%. Different genotype combinations led to varying probabilities of breast cancer, highlighting the importance of identifying those combinations with the highest predictive power and conferring the greatest susceptibility to breast cancer, serving as a foundation for further analysis.

**Table 2:** Parameter learning results of patients with or without breast cancer (partial).

Influence variable							Parameter learning results (breast cancer or not)	
rs1042522	rs1801133	rs1801394	rs1805087	rs56221660	rs7744	rs7851696	No	Yea
CG	A	A	A	A	A	G	0.0002	0.9998
C	AG	A	A	A	A	G	0.0001	0.9999
CG	AG	A	A	A	A	G	0.2727	0.7273
CG	G	A	A	A	A	G	0.9998	0.0002
G	G	A	A	A	A	G	0.9998	0.0002
CG	A	AG	A	A	A	G	0.9998	0.0002
C	AG	AG	A	A	A	G	0.2500	0.7500
CG	AG	AG	A	A	A	G	0.6667	0.3333
G	AG	AG	A	A	A	G	0.0001	0.9999
C	AG	A	GA	A	A	G	0.2000	0.8000
G	AG	A	GA	A	A	G	0.9998	0.0002
CG	G	A	GA	A	A	G	0.9998	0.0002
CG	AG	AG	GA	A	A	G	0.2000	0.8000
C	AG	A	A	AG	A	G	0.0001	0.9999
CG	AG	A	A	AG	A	G	0.9999	0.0001
CG	AG	AG	A	A	G	G	0.6666	0.3334
G	AG	AG	A	A	G	G	0.6666	0.3334
CG	AG	A	A	A	GA	G	0.5714	0.4286
G	AG	A	A	A	GA	G	0.4000	0.6000
C	AG	A	A	A	A	GT	0.2500	0.7500
C	G	AG	A	A	G	GT	0.6666	0.3334

it is indicated that these extreme probabilities reflect characteristics of the sample distribution rather than clinical predictive value.

#### 3.3.3 Posterior probabilistic inference in Bayesian networks

Posterior probability in Bayesian networks represents the updated probability of an event after incorporating new evidence. In this study, posterior probability was used to estimate breast cancer risk based on specific SNP combinations. The BNT in MATLAB was employed to perform inference, using the linkage tree inference engine. The primary evidence variable was set to the presence or absence of breast cancer. Analysis explored all possible combinations of genotypes across the SNPs included in the model. With three potential categories per node and nine loci, there were 3^9^ = 19 683 potential genotype combinations. From these, the combination with the highest probability of association with breast cancer was identified (maximum likelihood interpretation). The optimal combination of genotypes associated with breast cancer presence was identified as {rs1042522 = C, rs17884306 = C, rs1801133 = AG, rs1801394 = A, rs1805087 = A, rs56221660 = A, rs7744 = A, rs7851696 = G, rs9651118 = CT}. Validation of this optimal combination using test data revealed a marked increase in the predicted prevalence of breast cancer. The prevalence under the original data was 48.20%, while the prevalence of the highest-risk combination observed in this small sample increased to 99.99% (**Table 3**). These results demonstrate the enhanced predictive accuracy achieved by identifying and incorporating high-risk genotype combinations, underscoring the value of Bayesian network modeling in elucidating breast cancer susceptibility.

**Table 3:** The highest-risk combination observed in the current small sample.

Distribution of raw breast cancer values	Distribution of breast cancer values under optimal combination
Disease-free 51.80%	Disease-free 0.01%
Disease 48.20%	Disease 99.99%

#### 3.3.4 Model performance evaluation

The performance metrics for the Bayesian network model constructed to predict female breast cancer susceptibility are presented in **Table 4**. The accuracy, precision, and recall rates of the model for diseased samples were 78.39%, 82.64%, and 72.46%, respectively, indicating effective recognition of breast cancer cases. Furthermore, the relatively close values among metrics indicates well-balanced model performance in data classification, with no significant bias toward any category, demonstrating its overall reliability for this dataset.

**Table 4:** Evaluation of model performance.

	Precision	Recall	F1-score
Disease-free	75.00%	84.44%	79.44%
Disease	82.64%	72.46%	77.22%
Accuracy			78.39%

### 3.4 External validation of the model

**Figure 5:**
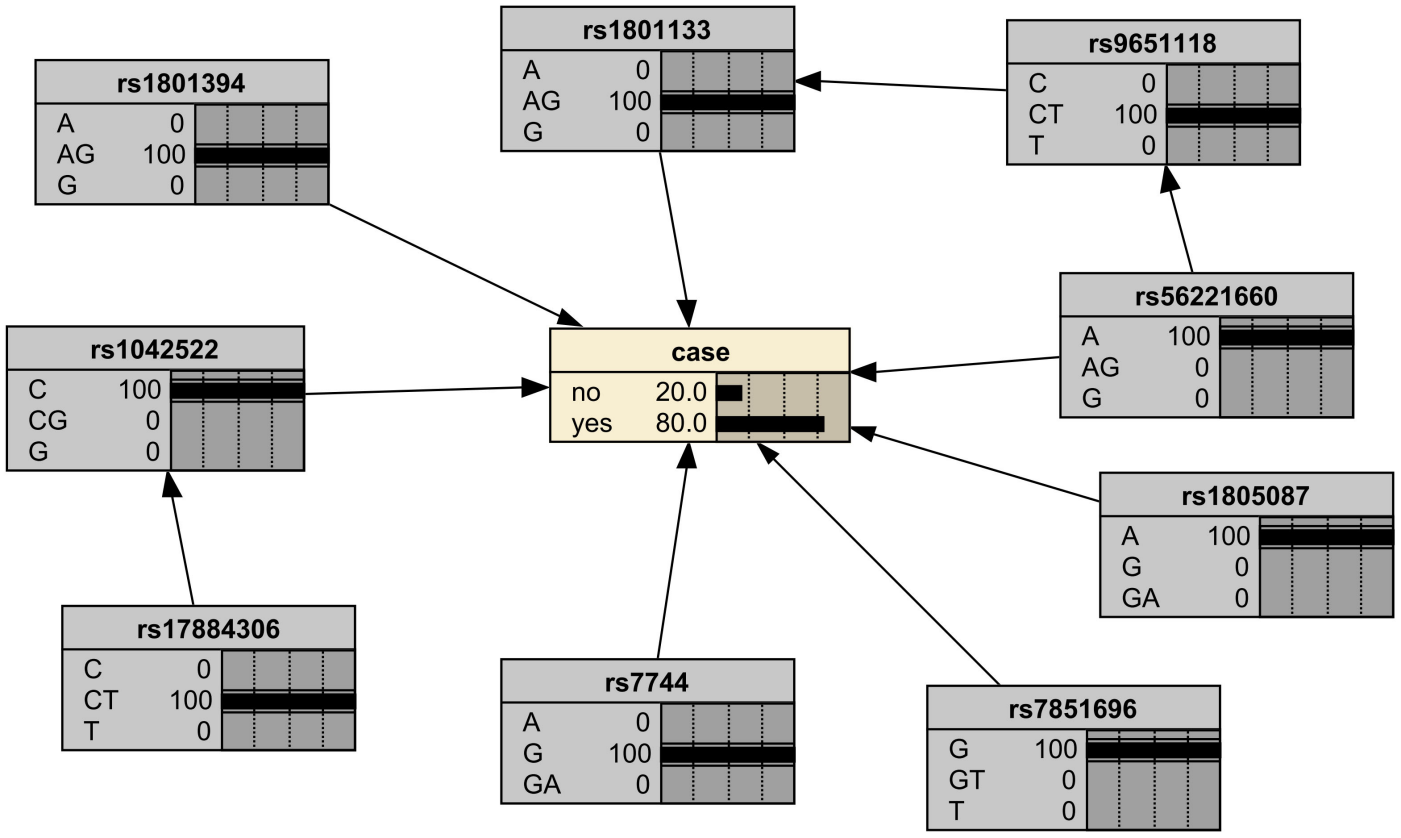
Bayesian network model validation.

To evaluate the effectiveness of the Bayesian network model, external validation was conducted using data from 10 case/control samples from the same hospital. The causal factors of each sample were input into the model to calculate the probability of breast cancer occurrence. For illustration, the genotype distribution of one sample diagnosed with breast cancer is provided: {rs1042522 = C, rs17884306 = CT, rs1801133 = AG, rs1801394 = AG, rs1805087 = A, rs56221660 = A, rs7744 = G, rs7851696 = G, rs9651118 = CT}. These genotypes were set as evidence variables and input into the Bayesian network model (**Figure 5**). The test results show that the model inferred an 80% probability of breast cancer for this patient, aligning closely with the clinical diagnosis. Similar analyses were performed for the remaining nine samples, and the model consistently demonstrated a 70% probability of correctly predicting breast cancer presence or absence. These research results demonstrate that the relationships among SNPS identified by Bayesian networks are consistent with the observed results, supporting the effectiveness of models constructed based on genotype data in predicting the risk of breast cancer.

## 4 Discussion

This study identified significant genetic loci associated with breast cancer development through both traditional statistical methods and machine learning approaches. Using chi-square tests, rs1801133 (*P* = 0.005) and rs56221660 (*P* = 0.049) were found to be statistically significant, suggesting their potential association with breast cancer susceptibility. The RF algorithm further analyzed 27 genetic loci, identifying eight key loci with feature importance scores exceeding 0.05, including rs7744, rs1042522, rs1801133, rs1801394, rs7851696, rs1805087, rs17884306, and rs9651118, deemed significant contributors to breast cancer development in women.

A Bayesian network model was subsequently constructed to investigate the probabilistic relationships between these loci and breast cancer prevalence. Based on parameter learning, the network assessed the probabilities of disease under various genetic combinations. In the original data, the prevalence and non-prevalence rates of breast cancer were 51.8% and 48.2%, respectively. This distribution failed to provide sufficient discriminative information. However, through Bayesian network posterior inference, we identified a maximum a posteriori (MAP) genotype combination: {rs1042522 = C, rs17884306 = C, rs1801133 = AG, rs1801394 = A, rs1805087 = A, rs56221660 = A, rs7744 = A, rs7851696 = G, rs9651118 = CT}. When this combination was incorporated into the model, the predicted prevalence of breast cancer increased to 99.99%, while the non-prevalence rate decreased to 0.01%. These results indicate that the risk of breast cancer increases significantly when the genotypes of key gene loci undergo specific changes. It should be noted that the results obtained from this MAP combination are based solely on the tests performed on the samples in this study. Under this MAP combination, there is an extremely high impact on the occurrence of breast cancer. This posterior probability combination is derived from sample-based search and learning, integrating the maximum impact of each locus on the development of breast cancer. In the future, if the dataset changes, this combination can still provide a reference for understanding the occurrence of breast cancer. This MAP combination successfully identifies genotype patterns highly associated with breast cancer, revealing the synergistic effects across multiple loci. (**Table 3**). These findings suggest that specific combinations of polymorphisms at these loci may have a profound influence on breast cancer development, highlighting the importance of multi-locus interactions in disease etiology. The results further underscore the complex interplay between genetic loci in the pathogenesis of breast cancer, implicating these specific SNPs as potential contributors to the underlying mechanisms of the disease. This study provides a basis for future functional studies to explore the roles of these loci in breast cancer. Additionally, the identified SNPs offer potential as molecular markers for early diagnosis and individualized treatment strategies, paving the way for more precise clinical interventions.

Model performance was evaluated by dividing the data into training and testing sets at a 7:3 ratio. The training set was used for model construction and parameter optimization, while the testing set was used to evaluate the generalization ability of the model. The model achieved an accuracy of 78.39%, a precision of 82.64%, and a recall of 72.46% for diseased samples, indicating its effectiveness in identifying breast cancer cases. These metrics suggest that the Bayesian network model is both reliable and generalizable, providing robust predictive power for breast cancer susceptibility in unseen data.

This study identified nine loci, including TP53 (rs1042522, rs17884306), MTHFR (rs1801133, rs56221660, 9651118), MTRR (rs1801394), MTR-A2756G (rs1805087), MYD88 (rs7744), and rs7851696, that may contribute to breast cancer susceptibility. Among these, mutations in TP53 are well-established risk factors for breast cancer development. As a critical regulator of nucleotide homeostasis, TP53 plays an important role in maintaining the nucleotide pool required for DNA synthesis and repair, thereby preserving genomic stability (34). The folate metabolism-related enzymes MTHFR and MTR are central to folate metabolism, and their enzymatic activities affect DNA methylation and synthesis. Impaired function of these enzymes can result in poor folate metabolism, reduced genomic stability (35), and increased susceptibility to cancer. Mutations in these loci are therefore closely linked to breast cancer progression. MYD88, a pivotal mediator in Toll-like receptor (TLRs)-initiated inflammatory cascades, is preferentially recruited to the Toll/interleukin-1 receptor (TIR) domain, which is conserved among specific TLR subtypes (36). Upon recruitment, MYD88 orchestrates the activation of the upstream nuclear factor-κB (NF-κB) kinase (inhibitor of kappa B kinase, IKK) complex (37), thereby serving as a central regulatory node in the activation of the NF-κB signaling pathway.​The rs7744 polymorphism, previously identified within the 3’-untranslated region (3’-UTR) of the MYD88 gene, shows significant association not only with treatment outcomes in rheumatoid arthritis (RA) (38) patients receiving tumor necrosis factor (TNF) inhibitors but also with disease progression in ulcerative colitis (UC) (39).​ Moreover, as a critical effector in inflammatory signaling cascades, aberrant expression or mutations of MYD88 correlate with poor prognosis in diffuse large B-cell lymphoma (DLBCL) (40).

Furthermore, MTRR, a key enzyme in cysteine metabolism, influences the production of hydrogen sulfide (H_2_S), a critical mediator in the NF-κB inflammatory pathway, through its effects on DNA methylation (13). This suggests a potential synergistic relationship between MYD88 and MTRR in activating the NF-κB inflammatory pathway, thereby contributing to cancer progression.

Bayesian network modeling is particularly effective when applied to dichotomous variables, as dichotomy minimizes the number of combinations and chances. In this study, the dichotomous classification—diseased versus non-diseased—allowed us to uncover a strong association between the combination of nine polymorphic loci and breast cancer susceptibility. Analysis suggested that enzymes involved in folate metabolism may synergize with inflammatory mediators, such as MYD88, to promote tumorigenesis. This interaction potentially disrupts folate metabolism, thereby impairing DNA methylation and synthesis, and simultaneously affects inflammatory pathways through the homocysteine cycle, driving tumorigenesis and cancer progression. The heterogeneity of breast cancer further complicates its genetic and phenotypic characterization, with distinct molecular subtypes classified based on the expression of key biomarkers: estrogen receptor (ERα), progesterone receptor (PR), human epidermal growth factor receptor-2 (HER-2), and proliferating cell nuclear antigen (Ki-67). The four widely recognized subtypes include luminal A, luminal B, human epidermal growth factor receptor-2 (Her-2) overexpression, and basal-like breast cancer (BLBC). Each subtype has distinct clinical implications and influences treatment strategies. For example, luminal A tumors respond well to endocrine therapy, while chemotherapy is often preferred for luminal B patients. HER-2-positive cases are likely to benefit from targeted therapies, whereas BLBC remains challenging due to its lack of validated therapeutic targets and poorer prognosis. Given these complexities, future research will focus on evaluating differences in Bayesian network structures and conditional probabilities across molecular subtypes of breast cancer. This approach aims to enhance the precision of treatment selection by tailoring strategies to the unique genetic and molecular characteristics of each subtype, ultimately improving patient outcomes.

The samples in this study were derived from Southwest China, primarily individuals who have resided in Yunnan Province for three or more generations. This population exhibits certain regional specificities in genetic background and environmental exposure profiles. Therefore, when extending the research findings to other regions, their broader applicability could be validated through multi-center studies incorporating diverse datasets. Regarding the sample size, although 490 breast cancer patients and 490 control subjects were included, the current sample size still has room for expansion in covering all potential genotype combinations (e.g., 19,683 combinations formed by 9 loci) when analyzing the complex interactions among multiple gene loci. This may, to some extent, affect the precision of statistical tests and the robustness of the results. For external validation, the model was tested using 10 independent samples. Its generalizability awaits further support from larger-scale independent datasets to more comprehensively evaluate its applicability across different scenarios. Additionally, attention should be paid to balancing the complexity of genotype combinations with the existing sample size. The analysis of interactions among multiple gene loci in this study involved complex probabilistic modeling, and the limited sample size may introduce potential estimation biases when identifying optimal genotype combinations. Thus, the strong associations observed in the training data (e.g., the 99.99% disease probability in the optimal combination) require further confirmation in larger samples to clarify their actual clinical relevance, which also provides directions for optimizing model parameters and enhancing result reliability and reproducibility.

However, it is undeniable that this study proposed an analytical framework of “predicting the whole from parts,” starting with investigating the impact of single gene polymorphisms on breast cancer probability and gradually extending to the combined effects of two, three, or more gene polymorphisms. This approach not only intuitively reflects the influence of individual gene polymorphisms (through probability changes) but also clearly demonstrates the association patterns between different gene polymorphisms. The application of the Bayesian network (BN) model to assist clinicians in prioritizing disease risk assessment holds practical value, as it can predict breast cancer diagnostic probabilities based on partial genotyping results of patients. The persuasiveness of the model will be further strengthened by continuously incorporating genetic polymorphism data from more confirmed breast cancer cases. The modular structure of the BN is inherently suitable for incremental learning and expansion, eliminating the need to rebuild the model from scratch when new data or variables are added—only adjustments to the network structure and parameters are required. This lays a methodological foundation for including more samples or gene loci in future studies, which is forward-looking in gene-disease association research.

## Data Availability

The raw data supporting the conclusions of this article will be made available by the authors, without undue reservation.
